# Not obtaining a medication the first time it is prescribed: primary non-adherence to cardiovascular pharmacotherapy

**DOI:** 10.1007/s00392-023-02230-3

**Published:** 2023-05-20

**Authors:** Martin Schulz, Ulrich Laufs

**Affiliations:** 1 Institute of Pharmacy, Freie Universität Berlin, Kelchstraße 31, 12169 Berlin, Germany; 2 Drug Commission of German Pharmacists (AMK), Heidestraße 7, 10557 Berlin, Germany; 3 German Institute for Drug Use Evaluation (DAPI), Heidestraße 7, 10557 Berlin, Germany; 4 Department of Cardiology, University of Leipzig, Liebigstraße 20, 04103 Leipzig, Germany

**Keywords:** Medication adherence, Primary non-adherence, Initial non-adherence, Cardiovascular disease, Statins, Antihypertensives

## Abstract

Primary medication non-adherence describes the situation when a first prescription for a new medication is never filled. Primary non-adherence is an important, yet understudied aspect of reduced effectiveness of pharmacotherapy. This review summarizes the frequency, impact, reasons, predictors, and interventions regarding primary non-adherence to cardiovascular/cardiometabolic drugs. The current literature reveals a high prevalence of primary non-adherence. The individual risk of primary non-adherence is determined on multiple factors, e.g., primary non-adherence of lipid-lowering drugs is higher compared to antihypertensive medications. However, the overall rate of primary non-adherence is > 10%. Additionally, this review identifies specific areas for research to better understand why patients forgo evidence-based beneficial pharmacotherapy and to explore targeted interventions. At the same time, measures to reduce primary non-adherence—once proven to be effective—may represent an important new opportunity to reduce cardiovascular diseases.

## Introduction

Cardiovascular diseases (CVD) remain the leading cause of death globally [1]. Pharmacological treatments can substantially reduce CVD morbidity and mortality. However, the effectiveness of these interventions is limited in cases of medication non-adherence and early discontinuation (non-persistence). Moreover in an aging population, polypharmacy is a growing problem in the elderly with 50% of adults above the age of 79 years using ≥ 5 chronic medications and nearly 20% taking ≥ 10 medications, which increases the risk of adverse drug effects (ADE) and drug–drug interactions (DDI), and decreases medication adherence [2–4].

The vast majority of medication adherence research focuses on identifying factors and outcomes associated with either issues of implementation (secondary) adherence, i.e., whether or not patients refill their prescriptions after the initial fill and takes the medication as agreed and prescribed, exploring the quality of the execution and/or non-persistence [5, 6], i.e., early discontinuation not intended by the prescriber [7, 8].

**Fig. 1:**
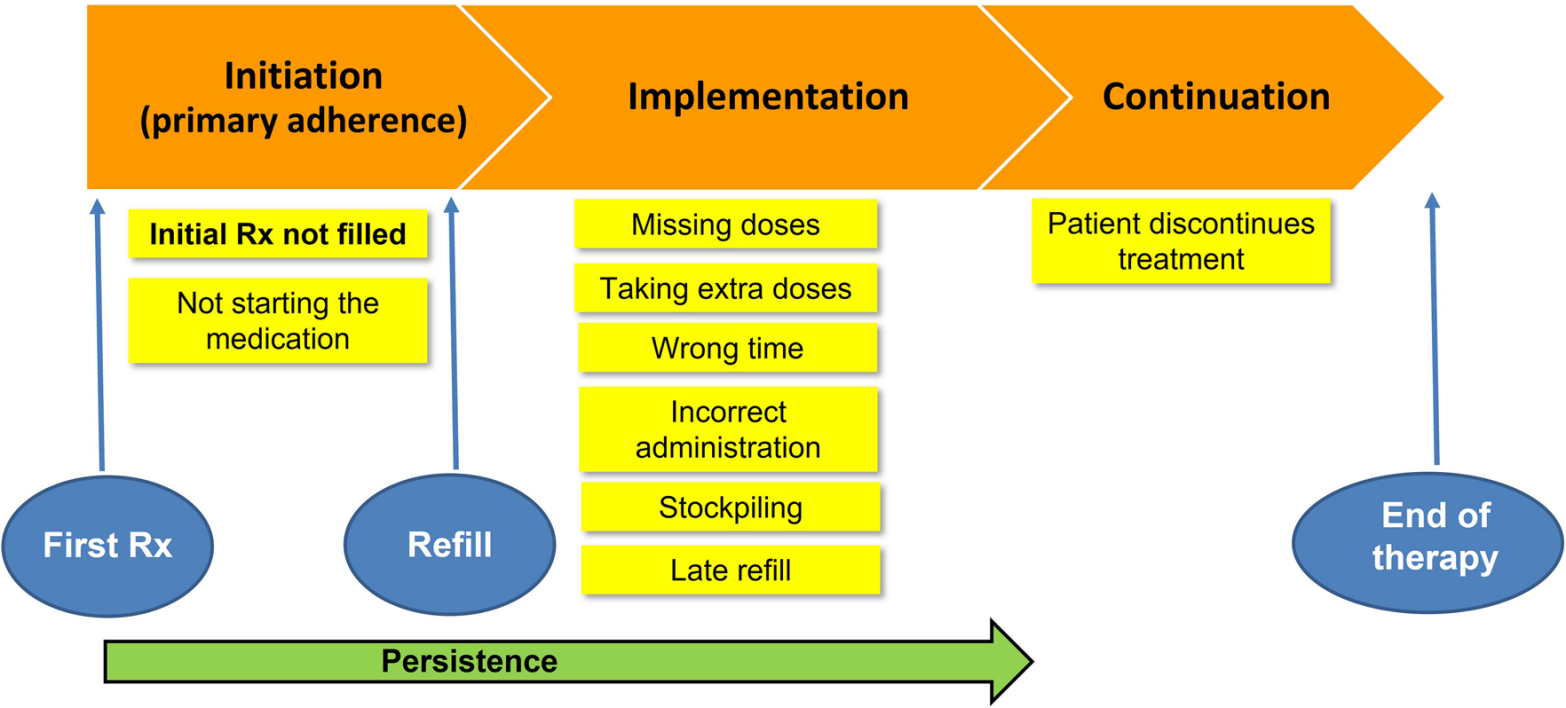
Conceptualization of medication adherence (modified according to [8–10]). *Rx* prescription

Primary medication non-adherence describes the situation when a provider prescribes (or orders) a new medication for a patient and the order is never filled or dispensed and the patient does not obtain an appropriate alternative within an acceptable time. In this scenario, the patient either never takes the prescription to the pharmacy or does take or sends the prescription to the pharmacy, but never comes back to pick up the medication. Furthermore, initiation can be divided into patients having a new prescription that is not followed by dispensing versus prescribed and dispensed medication that is not followed by commencement of treatment (Fig. 1) [8–11].

Advances in technology, including electronic prescribing and the development of electronic medical records, have facilitated more rigorous population-based studies of primary non-adherence [12, 13]. As an important limitation, these methodologies are not able to measure whether a new medication is handed out by a pharmacist to the patient, but the drug is not taken by the patient.

Specific measures to reduce primary non-adherence may have a potential impact on atherosclerotic cardiovascular diseases (ASCVD). A comprehensive review of the frequency, impact, reasons, predictors, and interventions regarding primary non-adherence to cardiovascular/cardiometabolic drugs is absent. Therefore, this paper aims to close this gap.

## Methods

We searched the bibliographic databases MEDLINE (via PubMed) and EMBASE (via Ovid) from their inception to 16th March 2023, using the terms “primary medication adherence”, “primary medication compliance”, “primary medication non-adherence”, “primary medication nonadherence”, “primary medication non-compliance, “primary adherence”, “primary compliance”, “primary non-adherence”, “primary nonadherence”, “primary non-compliance”, “baseline adherence”, “baseline compliance”, “baseline non-adherence”, “baseline non-compliance”, “initial adherence”, “initial compliance”, “initial non-adherence”, “initial nonadherence”, “initial non-compliance”, “initial medication non-adherence” or “initial medication nonadherence” without language restrictions. We further searched ClinicalTrials.gov for completed studies (no further restrictions) using the terms “primary OR initial medication adherence AND cardiovascular diseases”.

The search identified 204 articles, 49 in MEDLINE and 155 in EMBASE, and 430 registered studies in ClinicalTrials.gov. Titles and abstracts were screened for suitability regarding all types of cardiovascular/cardiometabolic drugs including lipid-lowering medication, anticoagulants, and hypoglycemics. Descriptions of the methodology used to measure primary non-adherence, articles on study design, short summaries, editorials, conference abstracts, and letters addressed to the editor were excluded. The search was supplemented by a hand search of the reference lists of all selected full-text articles. After removing duplicates, 39 primary studies and two meta-analyses [14, 15] were identified.

Descriptive statistical analysis and graphical illustration of the location and spread of estimates of primary non-adherence using boxplots were performed using IBM SPSS 28.

## Results

The overall prevalence of primary medication non-adherence is high. By conservative estimation, the rate is > 10% [6, 13–40].

A meta-analysis published in 2018 included 24 studies. The pooled primary non-adherence rate for the four chronic disease medications, antihypertensives, lipid-lowering drugs, hypoglycemics, and antidepressants, was 14.6% (95% confidence interval [CI] 13.1–16.2%). Among these medication classes, primary non-adherence was highest for lipid-lowering medications: 20.8% (95% CI 16.0–25.6%) [14].

A systematic review and meta-analysis published in 2019 included 5 randomized controlled trials (RCT), 26 cohort, and 2 cross-sectional studies (*n = *539,156). 31 studies (*n = *519,971) were used for the meta-analysis [15]. The prevalence of primary non-adherence was 17% (95% CI 15–20%). Pooled rates were highest in osteoporosis (25%, 95% CI 7–44%) and dyslipidemia (25%, 95% CI 18–32%), and lowest in diabetes mellitus (10%, 95% CI 7–12%) [15].

Fischer et al. analyzed 195,930 e-prescriptions of 75,589 patients treated by 1,217 prescribers in the first year of a community-based e-prescribing initiative in the USA. Of 82,245 e-prescriptions for new medications, 72% were filled. In multivariate analyses, medication class was the strongest predictor of adherence. Primary non-adherence was common for newly prescribed medications treating chronic conditions such as hypertension (28.4%), dyslipidemia (28.2%), and diabetes (31.4%) [13].

The overall rate of primary non-adherence among Danish residents in a GP setting was 9.3% with 4.7% for drugs addressing cardiovascular diseases. Polypharmacy (≥ 5 chronic drugs) and a diagnosis of ischemic heart disease were associated with higher rates of primary non-adherence. Older age and higher income were significantly associated with lower rates of primary non-adherence [19].

In a US health system cohort of 5146 patients newly prescribed a glucagon-like peptide-1 receptor agonist (GLP-1-RA) or a sodium–glucose co-transporter-2 inhibitor (SGLT2i), the overall incidence of 30-day primary non-adherence was 31.8%. This incidence rate was 29.8% and 33.6% among those initiating a GLP-1-RA and SGLT2i, respectively. Age ≥ 65 years (aOR 1.37, 95% CI 1.09–1.72), Black race vs. White (aOR 1.29), diabetic nephropathy (aOR 1.31), and hyperlipidemia (aOR 1.18) were associated with a higher odds of primary non-adherence. Female sex (aOR 0.86), peripheral artery disease (aOR 0.73), and having the index prescription ordered by an endocrinologist vs a primary care provider (aOR 0.76) were associated with lower odds of primary non-adherence [41].

A recent study in the Netherlands included 65,877 subjects who received 181,939 new drug prescriptions issued by general practitioners. Overall, primary non-adherence was 11.5%. Within drug classes that are frequently used in primary care, primary non-adherence was 9.9%. Primary non-adherence was lowest for thyroid hormones (5.5%), 8.3% for cardiovascular drugs, and highest for nonsteroidal anti-inflammatory drugs (11.8%) and 12.8% for proton pump inhibitors. Several factors were associated with primary non-adherence, such as having comorbidities (OR 1.46, 95% CI 1.37–1.56 for > 3 active diagnoses, compared to no active diagnoses) or reimbursement status (OR 2.78, 95% CI 2.65–2.92 for not reimbursed drugs compared to fully reimbursed drugs) [18].

A study in Canada aimed to determine adherence to thienopyridine therapy after stent implantation, factors associated with suboptimal adherence, and association of suboptimal adherence with mortality. They evaluated 5263 older patients (> 65 years) who received drug-eluting stents (DES) and 6081 older patients who received bare metal stents (BMS). Primary non-adherence was observed among 6.9% in the DES group and 7.1% in the BMS group that did not fill a single prescription of a thienopyridine within 1 year of stent implantation. Premature discontinuation occurred in a progressive manner, with 28% in the DES group and 34% in the BMS group discontinuing therapy by 6 months. For DES patients, primary non-adherence (hazard ratio [HR] 2.68, 95% CI 1.77–4.07), 12 months proportion of days covered < 80% (HR 2.39, 95% CI 1.67–3.43), and prematurely discontinuing therapy within 6 months (HR 2.64, 95% CI 1.60–4.35) were associated with an increased risk of death [42].

Overall, 10–11% of patients with nonvalvular atrial fibrillation are primarily non-adherent to initially prescribed oral anticoagulants [43, 44]. In a study in Spain, direct oral anticoagulant (DOAC) patients showed threefold higher odds of primary non-adherence compared with vitamin K antagonist (VKA, acenocoumarol in particular) patients. Primary non-adherence varied between DOACs, ranging from 5% for apixaban to 16% for rivaroxaban [43].

A recent study followed 12,257 DOAC patients. Of these, 10.4% were initially non-adherent; 12.8% for apixaban, 8.6% for dabigatran, and 10.8% for rivaroxaban. Patients aged ≥ 80 years showed lower odds of primary non-adherence compared to those aged < 65 years. A history of diabetes, hypertension, or stroke/transient ichemic attack was inversely associated with primary non-adherence [44].

Despite emphasis on efforts to prevent cardiovascular disease (CVD), 13–39% of people never fill a prescribed statin [45–48]. In a study, patients with primary non-adherence to statin medications (*n = *173) completed a self-administered cross-sectional survey that assessed their attitudes and beliefs related to primary non-adherence and to potential motivators for statin use [46]. Ninety-nine patients (57.2%) never filled their prescription, and 74 (42.8%) filled but never took any statin. Over half failed to initially inform their prescriber they might not take the statin. Patients expressed desire for alternate treatment plans such as diet and/or exercise (77.4%) or natural remedies/dietary supplements (72.3%). 56.6% of the patients worried about the possibility of statin dependence or addiction. 15.6% of the patients noted that they would not take a statin based solely on CVD risk estimates, and hence for primary prevention [46].

Although proprotein convertase subtilisin/kexin type 9 inhibitors (PCSK9i) were approved in 2015, their high cost has led to prescription restrictions or even prior authorization practices and use of PCSK9i in clinical practice has been low [49, 50]. Of patients receiving a prescription in the USA 2015/16, 47.2% ever received approval. Of those approved, 65.3% filled the prescription, resulting in 30.9% of those prescribed PCSK9i ever receiving therapy [49].

Table 1 summarizes the identified studies regarding primary non-adherence to cardiovascular/cardiometabolic drugs.

**Table 1:** Estimates of primary non-adherence to cardiovascular/cardiometabolic drugs ([14, 15], supplemented)

First author, year, study location	Medication class	Duration of follow-up	Primary non-adherence (%)	95% CI
Aznar-Lou et al. 2017, Spain [16]	Overall	1 month	17.6 (single dispensing: 53.6)	
	Antihypertensives (ACEi)		7.5 (single dispensing: 17.9)	7.3–7.7
	Lipid-lowering drugs (statins)		8.8 (single dispensing: 10.9)	8.6–9.0
	Hypoglycemics (insulins)		13.2 (single dispensing: 13.2)	12.5–14.0
Casebeer et al. 2009, USA [51]	Lipid-lowering drugs	4 months	43.2	39.9–46.4
Chan et al. 2004, Canada [14]	Antihypertensives	60 months	33.5	31.3–35.8
	Lipid-lowering drugs		71.0	68.8–73.1
Charlton et al. 2021, Spain [44]	DOACs	Variable according to treatment episode; at least 1 month	10.4	
	Apixaban		12.8	
	Dabigatran		8.6	
	Rivaroxaban		10.8	
Cheetham et al. 2013, USA [6]	Lipid-lowering drugs (statins)	3 months	15.4	14.9–15.9
Comer et al. 2015, USA [35]	Antihypertensives	21 months	34.0	
Derose et al. 2013, USA [52]	Lipid-lowering drugs (statins)	3 months	18.4	17.4–19.5
Fallis et al. 2013, Canada [53]	Miscellaneous (ACEi, beta blockers, statins, …)	1 month after discharge	24.0	
Fernandez et al. 2017, USA [29]	Hypoglycemics	2 months	4.6	
Fischer et al. 2010, USA [13]	Antihypertensives	12 months	19.5	19.0–19.9
	Lipid-lowering drugs		19.9	19.2–20.6
	Hypoglycemics		21.9	20.8–23.0
Fischer et al. 2015, USA [54]	Antihypertensives	0.5 months	3.3	2.6–4.2
	Hypoglycemics		6.4	4.0–9.5
Hempenius et al. 2023, The Netherlands [18]	Overall	1 month	11.3	
	ATC Group C		8.3	7.8–8.7
	Antihypertensives		6.9	6.4–7.4
	Statins		7.2	6.2–8.2
Jackevicius et al. 2008, Canada [55]	Antihypertensives	1 month	6.4	5.8–7.1
	Lipid-lowering drugs		5.2	3.7–7.0
	Hypoglycemics		13.7	8.6–20.4
Jackson et al. 2014, USA [56]	Antihypertensives	12 months	11.3	
	Lipid-lowering drugs		12.4	
	Hypoglycemics		12.9	
Kardas et al. 2020, Poland [28]	Overall	1 month	20.8	
	ATC Group C		17.2	
	Diuretics		18.6	
	Beta blockers		17.1	
	CCB		18.0	
	ACEi		15.1	
	ARB		17.2	
	Statins		17.5	
	Atorvastatin		18.9	
	Simvastatin		14.3	
	Rosuvastatin		17.5	
	DOACs		20.2	
	Dabigatran		29.3	
	Rivaroxaban		16.6	
	VKA		15.7	
Karter et al. 2009, USA [57]	Antihypertensives	6 months	3.2	
	Lipid-lowering drugs		8.5	
	Hypoglycemics		4.0	
Karter et al. 2018, USA [58]	Antihypertensives	2 months	3.2	2.9–3.5
	Lipid-lowering drugs		8.5	7.8–9.2
	Hypoglycemics		4.0	3.6–4.5
Kerner et al. 2017, USA [59]	Antihypertensives	1 month	22.2	2.8–60.0
Ko et al. 2009, Canada [42]	Thienopyridines	12 months	7.0	
Lee et al. 2016, USA [60]	Lipid-lowering drugs	3 months	10.4	
Leguelinel-Blache et al. 2015, France [61]	Antihypertensives	8 months	4.5	
	Lipid-lowering drugs		1.4	
	Hypoglycemics		1.9	
Liberman et al. 2010, USA [62]	Lipid-lowering drugs	2 months	34.0	
Linnet et al. 2013, Iceland [63]	Overall	1 month	6.2	
	Antihypertensives		7.2–9.9	
	Hypoglycemics		7.7–9.8	
Lyles et al. 2016, USA [64]	Antihypertensives	6 months	Combined: 6.0	
	Lipid-lowering drugs			
	Hypoglycemics			
Luo et al. 2022, USA [41]	GLP-1-RA	1 month	29.8	
	SGLT2i		33.6	
McHorney et al. 2011, USA [65]	Antihypertensives	24 months (est.)	2.0	
	Lipid-lowering drugs		4.9	
	Hypoglycemics		1.8	
Navar et al. 2017, USA [49]	Lipid-lowering drugs (PCSK9i)	12 months	34.7	
O’Connor et al. 2014, USA [66]	Overall	2 months	13.3	11.9–14.7
	Anthypertensives		17.0	
	Lipid-lowering drugs		18.1	
	Hypoglycemics		12.4	
Pottegard et al. 2014, Denmark [19]	ATC Group C	4 months	4.7	
	ACEi		3.3	
	ARB		2.5	
	Statins		6.2	
	Hypoglycemics		4.0	
Raebel et al. 2012, USA [67]	Antihypertensives	1 month	7.0	6.3–7.8
	Lipid-lowering drugs		12.6	11.6–13.6
	Hypoglycemics		11.3	9.8–13.0
Rodriguez-Bernal et al. 2018, Spain [43]	OAC	Not reported	5.6 (VKA 4.3; DOACs 10.8)	
	Acenocoumarol		4.2	3.9–4.5
	Warfarin		10.9	7.1–16.6
	Apixaban		5.0	3.0–8.3
	Dabigatran		7.9	6.9–9.2
	Rivaroxaban		15.5	13.8–17.4
Shah et al. 2009, USA [68]	Hypoglycemics	1 month	15.0	13.0–17.2
Shah et al. 2009, USA [69]	Antihypertensives	1 month	17.1	15.8–18.5
Shin et al. 2012, USA [70]	Antihypertensives	3 months	7.8	7.5–8.0
	Lipid-lowering drugs		22.3	21.8–22.9
	Hypoglycemics		12.6	12.0–13.1
Singer et al. 2022, Canada [71]	Antihypertensives	3 months	30.3	
	Lipid-lowering drugs		15.2	
	Hypoglycemics		21.2	
Tamblyn et al. 2014, Canada [72]	Hypoglycemics	9 months	29.1	26.3–32.1
Thengilsdottir et al. 2015, Iceland [73]	Statins	1 year	6.3	5.2–7.3
Trinacty et al. 2009, USA [74]	Hypoglycemics	1 month	10.0	8.7–11.5
Vilaplana-Carnerero et al. 2020, Spain [17]	ACEi	3 months	5.7 (single dispensing: 18.4)	
	Antiplatelets		9.1	
	Statins		6.7 (single dispensing: 10.6)	
	Insulins		7.8	

*ACEi* angiotensin-converting enzyme inhibitors, *ARB* angiotensin receptor blockers, *ATC Group C* Anatomical Therapeutic Chemical classification system-Cardiovascular System, *CCB* calcium channel blockers, *DOAC* direct oral anticoagulants, *est.* estimated, *GLP-1-RA* glucagon-like peptide-1 receptor agonists, *OAC* oral anticoagulants, *PCSK9i* proprotein convertase subtilisin/kexin type 9 inhibitors, *SGLT2i* sodium–glucose co-transporter-2 inhibitors, *VKA* vitamin K antagonists

Considering the variability of studies (and their results) regarding different healthcare systems (e.g., countries or insurance plans with high vs. minor out-of-pocket drug costs), data sources, length of follow-up, and the impact of duplicate prescriptions, one can debate the precision of the estimates presented and their generalizability across therapeutic classes and patient populations.

**Fig. 2:**
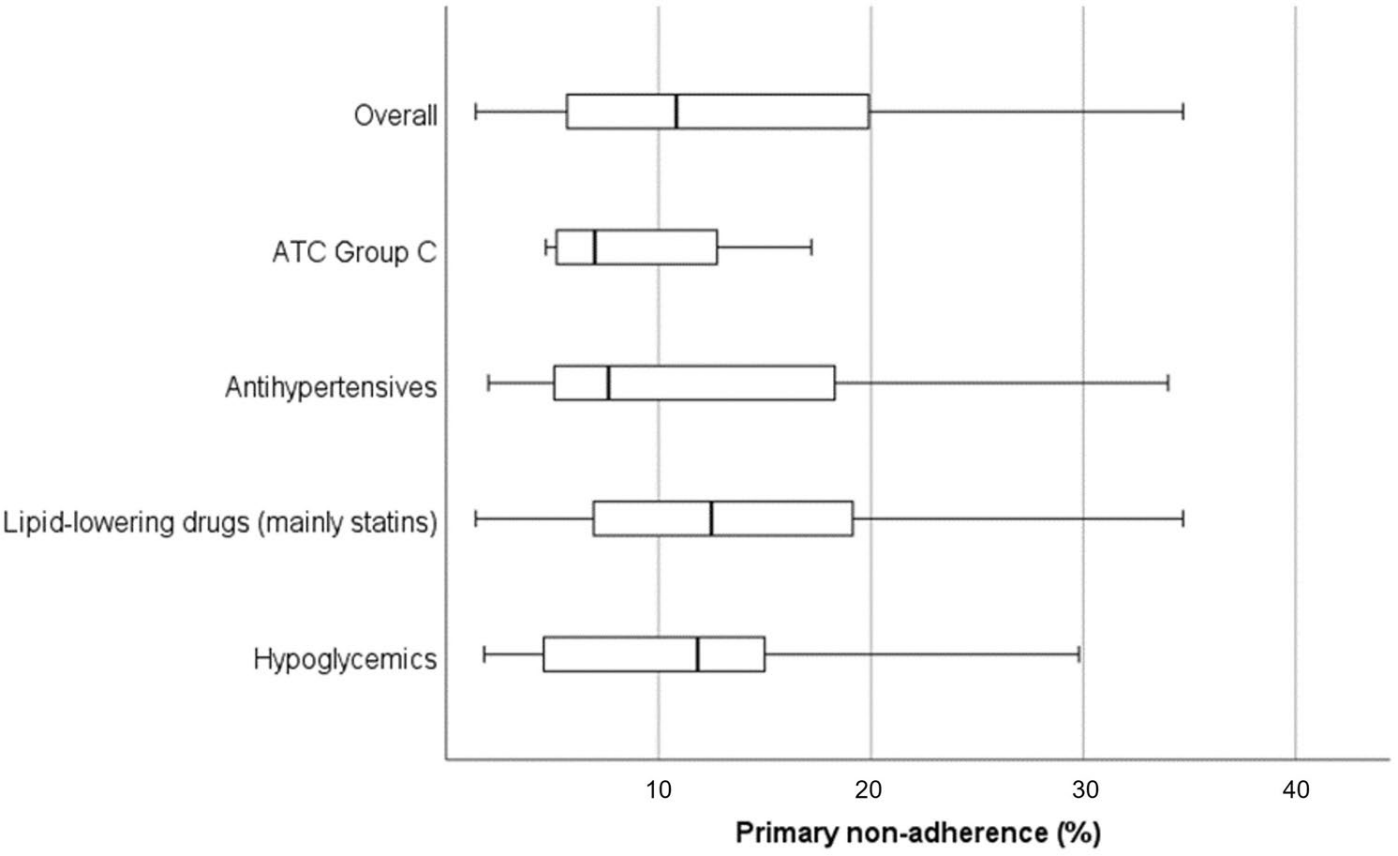
Boxplots of estimates of primary non-adherence to cardiovascular/cardiometabolic drugs. Outliers are not plotted [for citations of studies included, see Table 2]

However, it is apparent that the overall primary non-adherence rate is > 10% [14, 15]. We found a median of 10.9% (IQR 5.7–19.9%) and a somewhat lower rate for the entire ATC Group C (cardiovascular system) with a median of 7% (IQR 5.2–12.8%), based, however, on four publications only. Primary non-adherence rates for lipid-lowering drugs (mainly statins) and hypoglycemics are estimated higher with medians of 12.5% (IQR 7.0–19.2%) and 11.9% (IQR 4.6–15.0%), respectively (Table 2 and Fig. 2).

**Table 2:** Descriptive statistical analysis of estimates of primary non-adherence to cardiovascular/cardiometabolic drugs

Medication class	Primary non-adherence (%)				
	Minimum	25th percentile (*Q*_1_)	Median (*Q*_2_)	75th percentile (*Q*_3_)	Maximum
Overall^a^	1.4	5.7	10.9	19.9	71.0
ATC Group C^b^	4.7	5.2	7.0	12.8	17.2
Antihypertensives^c^	2.0	5.1	7.7	18.3	34.0
Lipid-lowering drugs^d^ (mainly statins)	1.4	7.0	12.5	19.2	71.0
Hypoglycemics^e^	1.8	4.6	11.9	15.0	33.6

*ATC Group C* Anatomical Therapeutic Chemical classification system-Cardiovascular System

^a^[6, 13–19, 28, 29, 35, 41–44, 49, 51–74]

^b^[17–19, 28]

^c^[13–18, 35, 54–59, 61, 63–67, 69–71]

^d^[6, 13–19, 28, 49, 51, 52, 55–58, 60–62, 64–67, 70, 71, 73]

^e^[13–16, 19, 29, 54–58, 61, 63–68, 70–72, 74]

### Reasons for and predictors of primary non-adherence

Reasons for primary non-adherence are likely diverse, spanning economic, social, and medical dimensions [75]. They include drug costs or high co-payments and fear of side effects [15, 28, 46, 47, 56, 62, 63, 68, 73, 76, 77]. For statins, a misbalance between perceived risks, poor acceptance/understanding of CVD risk estimates, and doubts about the benefits of statins are key [45–48, 60].

Similarly, the predictors of primary non-adherence are diverse and only partially understood. Several authors discuss younger age, high number of comorbidities, polypharmacy, low income, or lack of social support [14–16, 18, 19, 28, 43, 69, 72, 78–80]. In addition, discharge to a nursing home [53] and asymptomatic conditions, [71] such as hypercholesterolemia [47, 48, 81, 82], compared to symptomatic diseases, e.g., anxiety, depression, infection [71, 83], long distance to a pharmacy [84], or paper compared to electronic prescriptions [43, 85], have been described to be associated with the risk of primary non-adherence.

### Consequences of primary non-adherence

We were unable to identify studies that quantitate the effect of primary non-adherence for long-term cardiovascular/cardiometabolic medication intake and for studies that correlate primary non-adherence with clinical events. The lack of published data on the clinical consequences of primary non-adherence identifies an urgent need for future research.

### Interventions to reduce primary non-adherence

An RCT tested an automated outreach program to increase primary non-adherence to statins. Adult members of a health plan with no history of statin use within the past year who did not fill a statin prescription after 1–2 weeks were passively enrolled. The intervention group (*n = *2606) received automated telephone calls, followed 1 week later by letters for continued non-adherence. The control group (*n = *2610) received no outreach. Statins were dispensed to 42.3% of intervention and 26.0% of control participants (*p* < 0.001). Differences in the frequency of statin dispensations persisted up to 1 year (*p* < 0.001) [52].

In contrast, in a population of patients who had not picked up new prescriptions (asthma, hypertension, diabetes, or hyperlipidemia) after three calls from the pharmacy, additional nurse-directed outreach did not improve primary non-adherence [54]. In another US-health system study, automated reminder calls had no effect on primary non-adherence. However, live calls from pharmacists decreased antihypertensive primary non-adherence significantly; the difference in abandoned prescriptions for antihypertensives was 6.9% (*p* < 0.0001), but only 1.4% for antihyperlipidemics (*p* > 0.1) [86].

Addressing long-term adherence rather than primary non-adherence only, a study found that the length of initial prescription for elderly patients after coronary angiography at hospital discharge was associated with long-term adherence. The strength of the association was consistent for each cardiovascular secondary prevention medication, i.e., angiotensin-converting enzyme inhibitor (ACEi)/angiotensin receptor blocker (ARB), beta blocker, and statin, even after adjusting for relevant clinical and sociodemographic factors. Most prescriptions at discharge covered less than 1 month. This may be based on a clinical assumption that short prescriptions encourage patients to attend early outpatient follow-up. Although early follow-up is essential to assess the patient and to address medication side effects, the vast majority of patients did have follow-up within 1 month, regardless of prescription length [87].

Observational studies suggest that post-myocardial infarction (MI) patients receiving longer initial prescriptions have greater long-term adherence to cardiovascular medications [88]. The first, non-randomized interventional study to evaluate an intervention to standardize discharge prescriptions to prolonged duration to improve long-term medication adherence among post-MI patients, however, failed to meet its primary outcome. This was probably due to limited intervention fidelity, particularly at the pharmacy level [89]. Although a 5.4% absolute increase in long-term medication adherence after 12 months post-implementation in the standardized prolonged discharge prescription forms plus education group was observed, this absolute difference was not statistically significant. The authors recommended that a similar intervention be implemented and evaluated in a larger population with fully electronic medical records coupled with policies to support the long-term dispensation of medications at the community pharmacy level [89].

Randomized studies testing the effects of more comprehensive interventions to reduce primary non-adherence are scarce. A rare example is the study of a complex multidisciplinary intervention involving patients in the decision-making process to improve primary non-adherence to cardiovascular disease and diabetes treatment in primary care patients (IMA-cRCT) [90, 91]. Results of the pilot phase identified opportunities for refining the intervention and improving the design of a planned cluster RCT to evaluate the effectiveness and cost-effectiveness of the intervention [92].

## Discussion

Many patients do not fill their initial prescription for a cardiovascular/cardiometabolic medicine. At least one in ten newly prescribed medications do not make their way to the hands of the patients. Since ‘drugs can’t work if patients don’t receive them’ [42, 55, 60], primary non-adherence is a major healthcare problem. In addition to the negative impact on individual health, primary non-adherence is associated with a higher economic burden to the system in the short term, mainly due to higher productivity losses [93].

Most studies (62%) assessed primary non-adherence by following up (electronic) prescriptions for a 3-month duration or less. A sensitivity analysis of different durations (90 and 365 days) for defining primary non-adherence showed similar results to those when 30 days were applied [18].

Pottegard et al. found that 65.2% of the patients redeemed their prescription for a new drug on the same date that the prescription was issued and that 89.3% of the patients redeemed their prescription by day 30. Most of the patients filled their prescriptions within the 1st week. In another study analyzing new medication orders, 75% of new prescriptions were filled within 7 days and 81% within 30 days [33].

Prescriptions at the expense of the German Statutory Health Insurance (SHI) system (insuring approximately 88% of the total population—that is, 73.3 million people) are valid for 28 days after the date of issue. Private prescriptions are usually valid for 3 months after the date of issue.

96% of SHI prescriptions issued at hospital discharge in Germany between 2018 and 2021 were redeemed within three working days. However, these were mainly drugs indicated for a short period after discharge, e.g., analgesics/antiphlogistics, heparins, proton pump inhibitors, or anti-infectives. Long-term medications such as ramipril, bisoprolol, apixaban, or atorvastatin were prescribed less frequently at discharge (own unpublished data).

The review of the literature reveals a very heterogeneous set of data. For example, some of the observed primary non-adherence may reflect a wait-and-see approach [5]. A patient with a first prescription for a statin and borderline LDL-cholesterol level and atherosclerotic risk may first seek to control LDL-cholesterol by dietary changes and increased exercise leading to weight loss, eventually. Another reason for primary non-adherence to chronic medications is starting a new treatment (‘another pill’) is a long-term, sometimes lifelong commitment. Patients need understanding of the nature of the medical problem as well as the pros and cons of (not) treating it with the most effective and safe medication selected. When out-of-pocket costs are considerable or health insurance is absent at all, financial consequences play a major role in primary non-adherence [5, 14, 15, 58].

Primary non-adherence varied across therapeutic areas, drug classes, treatment duration, and individual drugs. One may expect lower primary non-adherence rates for conditions that present symptomatically (e.g., pain, anxiety, depression) or require short-term treatment (e.g., bacterial infections) compared to asymptomatic conditions with lifelong treatment indication (e.g., primary prevention of CVD). In fact, in one study, primary non-adherence ranged from 13.7% (antidepressants) to 17.5% (antibiotics) compared to 21.2% (hypoglycemics) to 30.3% (antihypertensives) for medications related to asymptomatic conditions [71].

Pottegard et al. found a lower primary non-adherence rate for *β*-lactam antibiotics (3.2%) and tramadol (5.2%), but not for nonsteroidal anti-inflammatory drugs (NSAIDs, 9.1%) [19]. Hempenius et al. found the highest primary non-adherence rates, defined as not having a prescription dispensed within 30 days from the prescription date, for proton pump inhibitors (12.8%) and NSAIDs (11.8%) compared to 8.3% for cardiovascular drugs [18].

Aznar-Lou et al. found an overall primary non-adherence rate of 17.6%. The rate was less for penicillins (9.8%), but higher for propionic acid derivatives (21.2%, such as ibuprofen) or anilides (22.6%, such as paracetamol) [16]. In a study in Poland, primary non-adherence was 20.8% overall, 17.2% for cardiovascular drugs and 14.3% for antibiotics [28].

One may conclude that not all new medications for symptomatic conditions are comparably acceptable to patients. Compared to anti-infectives, new prescriptions for pain medications (analgesics, NSAIDs) may be less likely to be filled.

Clinicians may have good reason to assume that after a confirmed diagnosis (e.g., for heart failure, hypertension, ASCVD), primary intervention (e.g., percutaneous coronary intervention (PCI)), or clinical event (MI, stroke) in a shared decision environment, the patient follows the agreed treatment plan. Often, the treatment starts with the initial prescription and obtaining the new medication at the pharmacy.

We are unaware of prospective studies comparing primary non-adherence in primary vs. secondary prevention of CVD. Moreover, the precise indication is rarely available in electronic prescription/dispensing records and, hence, typically not reported in the publications included in our review. Lipid-lowering medications (mainly statins) when compared to other cardiovascular drug classes had, however, the highest rate of primary non-adherence. A plausible explanation is that statins are often used for primary prevention. Studies show that primary prevention, among others, is a risk factor for secondary non-adherence (implementation and discontinuation, Fig. 1) to statins (rate ratio 1.52, 95% CI: 1.50–1.53) [94].

Similarly, the reported primary non-adherence to PCSK9i prescribed for patients with high risk for ASCVD is high [49]. In addition, approximately 7% primary non-adherence to thienopyridine therapy after stent implantation is concerning [42].

One problem is that primary non-adherence frequently goes undetected. The International Classification of Diseases (ICD) code Z91.1: “Patient’s noncompliance with medical treatment and regimen” is rarely used**.**

A potential reason is that clinicians are frequently unaware of the extent or consequences of non-adherence. In addition, complex factors surrounding medication non-adherence make it a challenge for the healthcare professional to identify and confirm non-adherence in general and primary non-adherence in particular [95]. Robust predictors for non-adherence to prescribed medication are absent and clinicians usually fail or significantly overestimate adherence behavior of ‘their’ patients (‘no better than a coin toss’) [96]. Providers recognized non-adherence for less than half of patients whose pharmacy records indicated significant refill gaps, and often intensified blood pressure medications even when suspected serious non-adherence [96, 97].

Hence, unidentified non-adherence may lead to unwarranted intensification of pharmacotherapy [95]. Medical education and training for screening, diagnosing, or treating non-adherence is rare [95, 98], but may represent an important opportunity to improve patient care.

Without access to electronic patient records including prescription and dispensing data, detection of primary non-adherence remains subject to trustful patient–provider communication [97]. Occasionally, screening of blood plasma, urine, or saliva for antihypertensives is needed to confirm the diagnosis ‘(primary) non-adherence’ (and, for example, ‘resistant’ hypertension) and to justify an intervention such as irreversible renal denervation [99–102].

Future research in different healthcare systems is needed to better understand why patients forgo evidence-based beneficial pharmacotherapy from the start and the clinical consequences of these decisions. We also need to improve our knowledge of detecting primary non-adherence and the efficacy and effectiveness of timely follow-ups and (digital) reminders [5, 103] or community pharmacy-based or pharmacist-led interventions [54, 61, 104–106]. To our knowledge, a digital (eHealth) application addressing primary non-adherence is not available.

Of note, apart from poor medication adherence, therapeutic/guideline inertia [107, 108] is independently associated with persistent poor blood pressure, LDL-cholesterol, or HbA1c control [109]. Therapeutic inertia may have a higher impact on disease control compared to medication adherence [109].

## Conclusions

Many patients fail to fill an initial prescription for a medicine. The rate of non-initiation of chronic cardiovascular/cardiometabolic medication is estimated to be > 10%. The true prevalence of single dispensation of a new medication is not extensively studied, but is most likely higher. Research is needed to better understand why patients forgo evidence-based beneficial pharmacotherapy and to explore targeted interventions. At the same time, measures to reduce primary medication non-adherence—once proven to be effective—may represent an important new opportunity to reduce cardiovascular diseases.

## Data Availability

Not applicable.
